# Tracing the decision-making process for myelofibrosis: diagnosis, stratification, and management of ruxolitinib therapy in real-word practice

**DOI:** 10.1007/s00277-019-03847-z

**Published:** 2019-12-12

**Authors:** Massimo Breccia, Claudia Baratè, Giulia Benevolo, Massimiliano Bonifacio, Elena Maria Elli, Paola Guglielmelli, Margherita Maffioli, Alessandra Malato, Francesco Mendicino, Giuseppe Alberto Palumbo, Novella Pugliese, Elena Rossi, Elisa Rumi, Emanuela Sant’Antonio, Alessandra Ricco, Mario Tiribelli, Francesca Palandri

**Affiliations:** 1 Hematology, Department of Precision and Translational Medicine, Policlinico Umberto 1, Sapienza University, Rome, Italy; 2 Department of Clinical and Experimental Medicine, Section of Hematology, University of Pisa, Pisa, Italy; 3 Hematology, Città della Salute e della Scienza, Turin, Italy; 4 Department of Medicine, Section of Hematology, University of Verona, Verona, Italy; 5 Hematology Division and Bone Marrow Transplant Unit, ASST Monza, Ospedale San Gerardo, Monza, Italy; 6 Center of Research and Innovation of Myeloproliferative Neoplasms, AOU Careggi, University of Florence, Florence, Italy; 7 Hematology Unit, ASST Sette Laghi, Ospedale di Circolo, Varese, Italy; 8 Division of Hematology and Bone Marrow Transplant, Villa Sofia-Cervello Hospital, 90146 Palermo, Italy; 9 Hematology Unit, Department of Hemato-Oncology, Ospedale Annunziata, Via San Martino, snc -, 87100 Cosenza, Italy; 10 Dipartimento di Scienze Mediche, Chirurgiche e Tecnologie Avanzate “G.F. Ingrassia”, University of Catania, Catania, Italy; 11 Department of Clinical Medicine and Surgery, University of Naples Federico II, Naples, Italy; 12 Istituto di Ematologia, Università Cattolica del Sacro Cuore, Fondazione Policlinico Universitario A. Gemelli IRCCS, Roma, Italy; 13 Department of Hematology Oncology, Fondazione IRCCS Policlinico San Matteo, University of Pavia, Pavia, Italy; 14 Department of Oncology, Division of Hematology, Azienda USL Toscana Nord Ovest, Lucca, Italy; 15 Medical Genetics, University of Siena, Siena, Italy; 16 Department of Emergency and Organ Transplantation (DETO), Hematology Section, University of Bari, Bari, Italy; 17 Division of Hematology and Bone Marrow Transplantation, Department of Medical Area, University of Udine, Udine, Italy; 18 Institute of Hematology “L. and A. Seràgnoli”, Sant’Orsola-Malpighi University Hospital, Bologna, Italy

**Keywords:** Myelofibrosis, Therapy, Real-life practice, Ruxolitinib, Survey

## Abstract

The management of patients with myelofibrosis (MF) has dramatically changed since the introduction of ruxolitinib as a tailored treatment strategy. However, the perceptions about the use of this drug in clinical practice remain, at times, a matter of discussion. We conducted a survey about the diagnostic evaluation, prognostic assessment, and management of ruxolitinib in real-life clinical practice in 18 Italian hematology centers. At diagnosis, most hematologists do not use genetically or molecularly inspired score systems to assess prognosis, mainly due to scarce availability of next-generation sequencing (NGS) methodology, with NGS conversely reserved only for a subset of lower-risk MF patients with the aim of possibly improving the treatment strategy. Some common points in the management of ruxolitinib were 1) clinical triggers for ruxolitinib therapy, regardless of risk category; 2) evaluation of infectious risk before the starting of the drug; and 3) schedule of monitoring during the first 12 weeks with the need, in some instances, of supportive treatment. Further development of international recommendations and insights will allow the achievement of common criteria for the management of ruxolitinib in MF, before and after treatment, and for the definition of response and failure.

**Electronic supplementary material:**

The online version of this article (10.1007/s00277-019-03847-z) contains supplementary material, which is available to authorized users.

## Introduction

Primary myelofibrosis (PMF) is a chronic myeloproliferative neoplasm clinically characterized by progressive anemia, bone marrow fibrosis, and extramedullary hematopoiesis with splenomegaly and/or hepatomegaly. Myelofibrosis can also be the end stage of other myeloproliferative neoplasms, namely, polycythemia vera (PV) and essential thrombocythemia (ET) (post-PV MF/post-ET-MF [PPV/PET-MF]) [1–3]. Patients affected by MF have a diminished quality of life and a survival duration ranging from 21 (including pre-fibrotic disease) down to approximately 1.5 to 4 years for patients with higher-risk disease [4–9]. Until recently, the primary goal of therapy was the alleviation of symptoms, and conventional therapies were unable to affect the biology of the disease meaningfully. Ruxolitinib is the only JAK1/2 inhibitor available for the treatment of MF. It may reduce myeloproliferation (through JAK2 inhibition) and the proinflammatory state (through JAK1 inhibition) associated with MF. This therapeutic effect may result in improvement in the symptom burden and a reduction of splenomegaly in a significant proportion of patients, reverting cachexia and possibly prolonging survival [10]. Nonetheless, no significant anticlonal effect was demonstrated during RUX therapy [11].

Two prospective phase III trials showed the superiority of the drug as compared to placebo and best available therapy, establishing ruxolitinib as the most effective therapy for MF-related splenomegaly and symptoms [12, 13]. However, ruxolitinib is burdened by significant hematological toxicity and cannot be prescribed to patients with low platelet count (i.e., below 50 × 10^9^/L) that represents around 15% of the total MF population. Also, ruxolitinib is associated with an increased risk of potentially severe infectious complications and occurrence of second neoplasms, thus requiring particular surveillance [14–16]. More specifically, a significantly increased risk of herpes zoster virus (HZV) infection was observed in ruxolitinib-treated patients compared to the control group in 3 randomized trials, including patients with polycythemia vera, and in a pooled analysis of the extended phase IIIa trials [12, 13, 15, 17–20]. In a larger phase IV post-marketing, expanded access study that included 1144 patients, the incidence of the most frequent infections was 8% for HZV, 6.1% for bronchitis, and 6% for urinary tract infections [17]. The most frequent atypical infections were tuberculosis, hepatitis B virus (HBV) infection reactivation, and *Pneumocystis jirovecii* infection. In a recent retrospective analysis on 446 patients treated outside clinical trials, 123 experienced 161 infectious episodes; the rate tended to decrease over time with respiratory tract infections being the most frequently observed [15]. Also, ruxolitinib therapy may be associated with an increased risk of second neoplasia, including non-melanoma skin cancer and lymphomas [21–23].

A number of retrospective studies have contributed to clarifying the management of ruxolitinib in real-life practice, although a clear picture of what happened outside of clinical trials since the commercial availability of ruxolitinib is still unknown. Also, there is a knowledge gap regarding the possible differential management of primary and secondary MF. The aim of this project was to record the perceptions of physicians about diagnostic evaluations, prognostic assessment, and disease management during ruxolitinib treatment. To this purpose, we created a specific online survey addressed to hematologists with clinical experience in myeloproliferative neoplasms.

## Methods

A project called “MPN Lab” was started in March 2018 with the aim of collecting experiences, perspectives, and proposals from 18 different Italian centers about the management of MF. The decision was made to conduct a survey among clinicians involved in the treatment of MF to gather more detailed and updated information on routine treatment practices and to identify further aspects of ruxolitinib use that pose challenges for physicians in the clinical practice. A survey with 41 questions was developed with closed answers about diagnosis of PMF and PPV/PET-MF, stratification with scoring systems (International Prognostic Scoring System or IPSS, Dynamic-IPSS or DIPSS, Molecular-IPSS or MIPSS, and Myelofibrosis Secondary to PV and ET-Prognostic Model or MYSEC-PM) [6, 7, 9, 24], the frequency of monitoring visits according to risk stratification, when and how to start a treatment according to baseline risk, routine research of non-driver mutations and cytogenetics, and the management of patients treated with ruxolitinib (evaluation of safety and efficacy). (The full survey questionnaire, including responses, is shown in Online Resource 1: Supplementary Table 1.) The survey circulated via the web, and the final results were collected by an external agency. The results were then discussed in the context of a meeting. Here we present the results summarized by descriptive statistics.

## Results

### Characteristics of participants

The participants (clinicians practicing in hospital settings) were selected from the entire Italian geographical territory and therefore may be considered to represent northern, central, and southern Italy and the islands. Also, the sample included both hematologists with extensive experience in MPNs and those with less experience (< 10 diagnosis of MF per year and/or < 10 years of clinical experience) and results therefore quite representative of the Italian reality.

### Initial diagnostic and prognostic evaluations

All participants reported adherence to 2008 World Health Organization (WHO) criteria for PMF [25]. While cytogenetic analysis was considered by most clinicians an important diagnostic tool, 18.8% declared that they reserved this analysis only for younger patients (< 50 years). Also, spleen size at diagnosis was assessed by palpation by all clinicians and confirmed by echography in less than half of the cases (43.8%). In real-life practice, magnetic resonance imaging (MRI)/computed tomography (CT) scans were never used at diagnosis.

Conversely, most physicians did not fully comply with the proposed criteria for PPV/PET-MF diagnosis. Indeed, a possible MF evolution from PV was evaluated with a new bone marrow biopsy mainly in the case of onset or progression of splenomegaly (68.8%), while the absence of phlebotomies requirement was taken into consideration only by 6.3% of the participants. Also, the main trigger for PET-MF diagnosis was the occurrence of splenomegaly combined with no treatment requirement in most cases (68.8%). Presence and/or worsening of systemic symptoms was never included among factors associated with ET/PV evolution into MF.

At diagnosis, all participants routinely used IPSS in PMF, while the MYSEC-PM was used in post-PV/ET MF by 81% of the physicians. The DIPSS was calculated by 93.7% of clinicians during the follow-up. A molecular laboratory equipped for testing non-driver mutations was available in-house in 37.5% of the centers, and only 25% of all participants routinely tested the MIPSS-70 or the plus versions. A minority of physicians (12%) tested non-driver mutations in all patients (regardless of the positivity of driver mutations), whereas 25% believed that next-generation sequencing (NGS) analysis was appropriate in younger patients (< 50 years); 37% of the physicians reserved the testing for high-molecular-risk (HMR) mutations only to intermediate-1 risk patients in order to possibly include allogeneic stem cell transplantation in the therapeutic algorithm. The remaining physicians (25%) did not test non-driver mutations in any instance.

The monitoring schedule was influenced by IPSS category. In 62% of the centers, visits were scheduled every 3 months for patients with low/intermediate-1 IPSS risk, while 50% scheduled the visits every month in patients at intermediate-2/high IPSS risk.

### Management of patients before ruxolitinib treatment

Physicians were first asked to state the main reason for starting ruxolitinib treatment in lower-risk categories. In intermediate-1 IPSS risk patients, ruxolitinib was started only in the case of splenomegaly palpable at least 5 cm below the left costal margin and concomitant significant burden of symptoms, evaluated as a score > 40 on the 10-item Myeloproliferative Neoplasm Symptom Assessment Form Total Symptom Score (MPN-SAF TSS) or as an elevated score in at least one item [26], in 93.7% of centers. The majority of participants (75%) evaluate symptom burden with the MPN-SAF TSS questionnaire. While the remaining hematologists reported treating intermediate-1 patients even in case of splenomegaly alone, none stated that they started treatment solely on the basis of MPN-SAF TSS; notably, no consensus was found on the exact cutoff criteria of the worst single symptom required for identifying patients who will most benefit from symptom-based treatment. Conversely, the main reason for starting ruxolitinib in low-risk patients was the presence of systemic symptoms (intended as MPN-SAF TSS > 0, regardless of severity) (37% of participants) and new onset of any-grade splenomegaly (31%). Twenty-five percent of the clinicians stated that ruxolitinib was never reserved for low-risk patients in their hematology centers, and 6% pointed out that a switch to higher-risk category was required for inclusion of ruxolitinib in the therapeutic algorithm. Notably, while 12.5% of the participants reported that, in their experience, more than 50% of low/intermediate-1 IPSS risk patients are symptomatic, the majority of the clinicians (62.5%) estimated this percentage to be lower than 30%.

In intermediate-2/high DIPSS patients with splenomegaly, ruxolitinib was used as frontline therapy by most doctors (87.5%), while the remaining considered ruxolitinib only after hydroxyurea failure.

Before starting ruxolitinib, 87% of participants declared evaluation of a complete panel of serological markers for hepatitis and, in the case of previous contact with HBV, to undertake prophylaxis with lamivudine. All participants except one also routinely investigated a previous tuberculosis infection. Tuberculosis screening was performed through interferon gamma release (QuantiFERON) assay in most (82%) cases, while 12% utilize the Mantoux intradermal test; in one of the centers, a chest X-ray was also routinely performed before the start of ruxolitinib. Only a minority of physicians (13%) reported routinely performing serological tests for all herpes infections including cytomegalovirus infection (CMV), herpes simplex virus (HSV), HZV, and Epstein-Barr virus (EBV), while an additional 13% performed only HSV tests. While all centers agreed not to initiate primary prophylaxis against herpes viruses, secondary prophylaxis was performed in the event of frequent herpes reactivations before ruxolitinib therapy or after the first episode of viral infection during treatment. In case of infections, ruxolitinib schedule was temporarily reduced by 62% of respondents, whereas 37.5% discontinued the drug and then later restarted at the same dose. Ruxolitinib discontinuation was unanimously considered temporary, and drug rechallenge was performed by all participants at the previously assessed maximum tolerated dose, without dose reductions due to the infectious episode.

### Management of patients during ruxolitinib treatment

During ruxolitinib therapy, monitoring was very variable, according to baseline hematology values. The majority of clinicians scheduled the visits weekly for the first 2–3 months or accordingly to hematological toxicity, whereas 18.7% of participants follow the patients every 2 weeks and 25% every month. Conversely, management of ruxolitinib-induced severe (grade ≥ 3) anemia was homogeneous among centers: 81% of physicians declared that the same dose was continued, supporting the patients with red blood cell transfusions, while the ruxolitinib dose was reduced by 19% of the clinicians. The use of erythropoietin was disclosed by 62% of the hematologists, regardless of endogenous erythropoietin (EPO) levels in 25% of cases. In the case of grade ≥ 3 thrombocytopenia during treatment, only 19% of clinicians reported discontinuing the therapy, while the great majority reduced the drug daily dose. In the case of ruxolitinib discontinuation, 81% of physicians gradually tapered the dose in order to avoid a withdrawal syndrome.

**Fig. 1:**
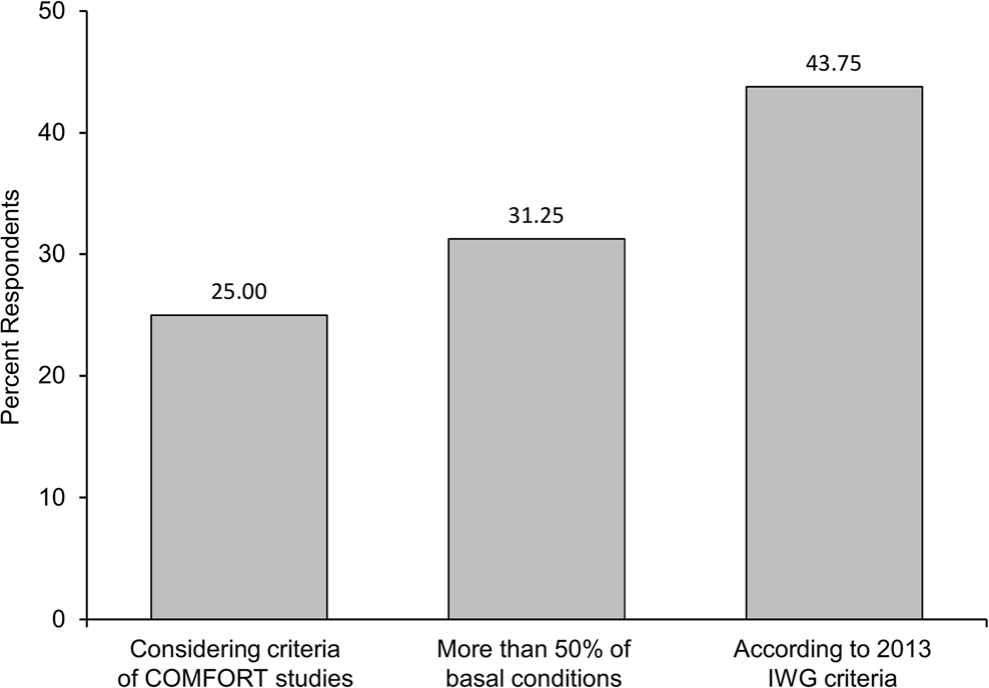
Criteria used by the respondents to evaluate splenomegaly

After starting the drug, physicians expected to observe an improvement of symptoms within 1–2 weeks (63%) or within 4 weeks (37%); delayed symptom responses were considered extremely rare. Notably, 75% of interviewed physicians used the MPN-SAF TSS 10-item questionnaire to evaluate the degree of symptom response. Conversely, there was no agreement in the modality of evaluation of spleen response (Fig. 1): 43.7% of physicians generally used the International Working Group for Myeloproliferative Neoplasms Research and Treatment (IWG-MRT) 2013 criteria [27], whereas a spleen response was considered a reduction of more than 50% of spleen length from baseline by 31% of the physicians and a reduction of more than 35% of volume from baseline (COMFORT criteria) by 25% [12]. Importantly, in long-lasting spleen responders, a dose reduction was never considered, unless in the case of concomitant toxicity. In addition, 50% of the interviewed doctors declared that they evaluated a bone marrow biopsy in all patients with complete symptoms and spleen response, after at least 1 year from treatment start.

**Fig. 2:**
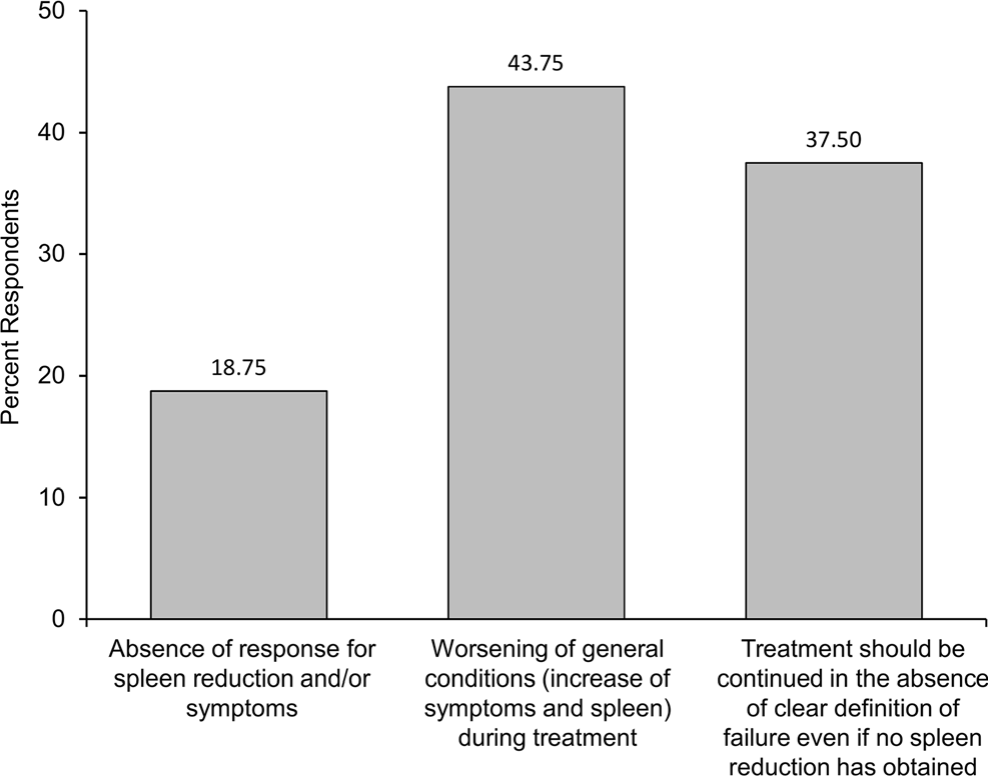
Definitions of failure of ruxolitinib treatment used by the respondents

The definition of failure of ruxolitinib therapy was also extremely discordant among participants (Fig. 2). Indeed, failure was defined as any worsening of clinical conditions (viz., symptoms and splenomegaly) by 43.8% of physicians and by lack of any spleen/symptom response by 18.8%. Notably, 37.5% of the hematologists declared that, in the absence of clear failure criteria, in their practice, ruxolitinib was generally continued over time depending only on tolerability, regardless of spleen response. In patients without optimal response or in those experiencing hematological toxicity, 81% of physicians would consider a possible combination of ruxolitinib with other agents. Hydroxyurea was frequently used to control splenomegaly and, especially, white blood cell count, whereas erythropoietin, danazol, and vitamin supplements are often used with the aim of mitigating anemia.

Finally, when asked about the role of allogeneic stem cell transplantation, most physicians would consider this option for intermediate-2/high IPSS risk at baseline and for intermediate-1 risk patients with additional negative prognostic factors (i.e., high-molecular-risk mutations), in substantial agreement with the European Society for Blood and Marrow Transplantation (EBMT) proposal [28].

A summary of therapy management of patients receiving treatment with ruxolitinib in Italy is presented in Table 1.

**Table 1:** Summary of therapy management of patients receiving treatment with ruxolitinib in Italy

**Well established in real-life practice**	
Adherence to World Health Organization diagnostic criteria for myeloproliferative neoplasms	
Preemptive screening for prior hepatitis B and C infection and latent tuberculosis	
Consideration of onset of splenomegaly in patients with suspected evolution to secondary myelofibrosis	
Routine use of ruxolitinib as frontline therapy in intermediate-2/high DIPSS patients with splenomegaly	
Use of secondary prophylaxis against herpes viruses in the event of frequent reactivations before or following the first episode of viral infection during treatment	
Use of DIPSS during follow-up	
Utilization of MYSEC-PM for secondary myelofibrosis	
**In need of improvement**	
More consistent use of IWG-MRT diagnostic criteria, cytogenic analysis, and/or echography scans for evaluation of spleen size	
Consideration of routine serological screening for all herpes virus infections	
Use of MPN-SAF TSS as a criterion for starting treatment in wider risk categories of patients	
More attention to systemic symptoms in patients with suspected evolution to secondary myelofibrosis, in particular, use of MPN-SAF TSS	
More uniform monitoring of ruxolitinib therapy, including scheduling of visits	
Coordination of criteria for the evaluation of spleen response and modality of evaluation	
Utilization of newer scores (MIPSS70, MIPSS70+, MIPSS70 + v2, and GIPSS) for secondary MF in patients other than younger low/intermediate-1 risk patients considered suitable for allogeneic stem cell transplantation	
Establishment of uniform criteria to define treatment failure, to include measurement of circulating peripheral blasts between 5 and 9%	

*DIPSS* Dynamic International Prognostic Scoring System, *GIPSS* Genetically Inspired Prognostic Scoring System, *IWG-MRT* International Working Group for Myeloproliferative Neoplasms Research and Treatment, *MPN-SAF TSS* Myeloproliferative Neoplasm Symptom Assessment Form Total Symptom Score, *MIPSS* Molecular International Prognostic Scoring System, *MYSEC-PM* Myelofibrosis Secondary to PV and ET-prognostic model

## Discussion

The aim of this survey was to explore the routine clinical behavior of Italian hematologists regarding diagnostic evaluations, prognostic assessment, and management of ruxolitinib therapy.

This study has some limitations. The limitation and potential bias relating to participation by researchers with some level of industry involvement were overcome by the wide-ranging discussion that involved all participating hematologists and concerned the entire “patient’s journey” (from diagnosis and prognostic evaluations that are not focused on ruxolitinib to the management of ruxolitinib outside clinical trials and including the definition of resistance). Indeed, in some cases, the response to the survey was amended after a second round of collegial discussion. The added value of the survey is to bring to light operational clinical behaviors, together with the difficulties and uncertainties that are unlikely to emerge from a sponsored clinical trial and/or retrospective observational studies.

The first result of this study is that prognosis is still assessed by clinical risk scores that do not require the evaluation of non-driver high-molecular-risk mutations. While the use of the MYSEC-PM for secondary MF seemed to have entered into standard clinical practice, newest scores (viz., MIPSS70, MIPSS70+, MIPSS70 + v2, and GIPSS [genetically inspired prognostic scoring system]) had not yet achieved widespread use, mainly due to poor feasibility (i.e., lack of molecular facilities) and financial concerns related to the costs of NGS analysis. Consequently, the assessment of high-molecular-risk mutations by NGS was reserved for a small fraction of younger low/intermediate-1 risk patients that may be suitable for allogeneic stem cell transplantation [8, 9, 29]. Also, spleen size is frequently assessed only by palpation, without radiological confirmation. This real-life practice, despite being rapid and inexpensive, obviously reduces the accuracy of this evaluation both at diagnosis and over the follow-up and should probably be reconsidered. Additionally, in the evaluation of patients with suspected evolution to secondary MF, the onset of splenomegaly was always taken into account, while the appearance of systemic symptoms was less frequently considered [3].

Regarding the management of ruxolitinib therapy, this survey disclosed three important results. First, there is a widespread consensus for preemptive screening for previous hepatitis B and C infection, as well as latent tuberculosis infections by the most sensitive QuantiFERON-TB Gold test. This attitude is in agreement with the recommendations of the European LeukemiaNet and the Italian Society of Haematology (ELN-SIE) guidelines that suggest specific monitoring and prophylactic measures in patients with at least one risk factor for infections [30, 31]. Second, not all physicians use the same criteria to evaluate spleen response, which was differently defined in terms of entity of reduction (from 35% to 50% and up to 100% in selected cases) and of the modality of evaluation (spleen length by palpation vs. spleen volume by echography). Third, physicians interviewed complained about the absence of uniform criteria for treatment failure. While the outcome of patients after ruxolitinib discontinuation has been reported to be poor [32–34], the definition of resistance was unclear. A recent Italian retrospective analysis of 218 MF patients who received and discontinued ruxolitinib therapy in routine clinical practice found that greater burden of disease at baseline is significantly associated with a higher discontinuation rate [35]. Accordingly, the appearance/increase of circulating peripheral blasts between 5 and 9% is associated with reduced survival in patients treated with ruxolitinib in chronic phase [36]. This parameter may be therefore included among features associated with ruxolitinib failure in future prospective trials.

A recent survey from the Gruppo Italiano Malattie EMatologiche dell’Adulto (GIMEMA) Myeloproliferative Neoplasms Working Party included 100 Italian hematology centers and documented some areas of uncertainties in the management of MF, including the limited use of echo-scan to assess spleen size, the limited assessment of IPSS at diagnosis, and the evaluation of HMR only in a very restricted subset of patients. Also, the MPN-SAF TSS score was routinely performed in less than 20% of the patients [37]. Compared to that survey, our real-life experience involved many hematologists with experience in the treatment of MF, who showed a more appropriate use of validated scores, both for MF diagnosis and for the assessment of symptom burden. Nonetheless, the reluctance in performing a radiological confirmation of the degree of splenomegaly and the difficulty in assessing HMR mutations even in transplant-eligible patients are common denominators of the two surveys and should be recognized as major areas for future improvement.

In conclusion, this survey has shown that many aspects of MF management, before and during ruxolitinib therapy, are well-established, at least from the point of view of the formal knowledge expressed by the respondents to the questionnaire (Table 1). Nonetheless, further improvements in the management of MF should be implemented. A common effort involving physicians, patients, and companies will allow the achievement of common criteria of management before and after treatment, response, and failure.

## Electronic Supplementary Material

Online Resource 1. Supplementary Table 1. Questionnaire and related answers. (PDF 174 kb)

## References

[1] Passamonti F (2012) Classification of myeloproliferative neoplasms and prognostic factors. Am Soc Clin Oncol Educ Book:419–424. 10.14694/EdBook_AM.2012.32.419 10.14694/EdBook_AM.2012.32.241 24451774

[2] Arber DA, Orazi A, Hasserjian R, Thiele J, Borowitz MJ, Le Beau MM, Bloomfield CD, Cazzola M, Vardiman JW (2016). The 2016 revision to the World Health Organization classification of myeloid neoplasms and acute leukemia. Blood.

[3] Barosi G, Mesa RA, Thiele J, Cervantes F, Campbell PJ, Verstovsek S, Dupriez B, Levine RL, Passamonti F, Gotlib J, Reilly JT, Vannucchi AM, Hanson CA, Solberg LA, Orazi A, Tefferi A, International Working Group for Myelofibrosis Research and Treatment (2008). Proposed criteria for the diagnosis of post-polycythemia vera and post-essential thrombocythemia myelofibrosis: a consensus statement from the International Working Group for Myelofibrosis Research and Treatment. Leukemia.

[4] Barosi G, Rosti V, Bonetti E, Campanelli R, Carolei A, Catarsi P, Isgrò AM, Lupo L, Massa M, Poletto V, Viarengo G, Villani L, Magrini U (2012). Evidence that prefibrotic myelofibrosis is aligned along a clinical and biological continuum featuring primary myelofibrosis. Plos One.

[5] Mesa RA, Jamieson C, Bhatia R, Deininger MW, Fletcher CD, Gerds AT, Gojo I, Gotlib J, Gundabolu K, Hobbs G, McMahon B, Mohan SR, Oh S, Padron E, Papadantonakis N, Pancari P, Podoltsev N, Rampal R, Ranheim E, Reddy V, Rein LAM, Scott B, Snyder DS, Stein BL, Talpaz M, Verstovsek S, Wadleigh M, Wang ES, Bergman MA, Gregory KM, Sundar H (2017). NCCN guidelines insights: myeloproliferative neoplasms, Version 2.2018. J Natl Compr Canc Netw.

[6] Cervantes F, Dupriez B, Pereira A, Passamonti F, Reilly JT, Morra E, Vannucchi AM, Mesa RA, Demory JL, Barosi G, Rumi E, Tefferi A (2009). New prognostic scoring system for primary myelofibrosis based on a study of the International Working Group for Myelofibrosis Research and Treatment. Blood.

[7] Passamonti F, Cervantes F, Vannucchi AM, Morra E, Rumi E, Pereira A, Guglielmelli P, Pungolino E, Caramella M, Maffioli M, Pascutto C, Lazzarino M, Cazzola M, Tefferi A (2010). A dynamic prognostic model to predict survival in primary myelofibrosis: a study by the IWG-MRT (International Working Group for Myeloproliferative Neoplasms Research and Treatment). Blood.

[8] Tefferi A, Guglielmelli P, Nicolosi M, Mannelli F, Mudireddy M, Bartalucci N, Finke CM, Lasho TL, Hanson CA, Ketterling RP, Begna KH, Naseema G, Pardanani A, Vannucchi AM (2018). GIPSS: genetically inspired prognostic scoring system for primary myelofibrosis. Leukemia.

[9] Guglielmelli P, Lasho TL, Rotunno G, Mudireddy M, Mannarelli C, Nicolosi M, Pacilli A, Pardanani A, Rumi E, Rosti V, Hanson CA, Mannelli F, Ketterling RP, Gangat N, Rambaldi A, Passamonti F, Barosi G, Barbui T, Cazzola M, Vannucchi AM, Tefferi A (2018). MIPSS70: mutation-enhanced international prognostic score system for transplantation-age patients with primary myelofibrosis. J Clin Oncol.

[10] Cervantes F, Pereira A (2017). Does ruxolitinib prolong the survival of patients with myelofibrosis?. Blood.

[11] Plosker GL (2015). Ruxolitinib: a review of its use in patients with myelofibrosis. Drugs.

[12] Verstovsek S, Mesa RA, Gotlib J, Levy RS, Gupta V, DiPersio JF, Catalano JV, Deininger M, Miller C, Silver RT, Talpaz M, Winton EF, Harvey JH, Arcasoy MO, Hexner E, Lyons RM, Paquette R, Raza A, Vaddi K, Erickson-Viitanen S, Koumenis IL, Sun W, Sandor V, Kantarjian HM (2012). A double-blind, placebo-controlled trial of ruxolitinib for myelofibrosis. N Engl J Med.

[13] Harrison C, Kiladjian JJ, Al-Ali HK, Gisslinger H, Waltzman R, Stalbovskaya V, McQuitty M, Hunter DS, Levy R, Knoops L, Cervantes F, Vannucchi AM, Barbui T, Barosi G (2012). JAK inhibition with ruxolitinib versus best available therapy for myelofibrosis. N Engl J Med.

[14] Palandri F, Tiribelli M, Benevolo G, Tieghi A, Cavazzini F, Breccia M, Bergamaschi M, Sgherza N, Polverelli N, Crugnola M, Isidori A, Binotto G, Heidel FH, Buccisano F, Martino B, Latagliata R, Spinsanti M, Kallenberg L, Palumbo GA, Abruzzese E, Scaffidi L, Cuneo A, Cavo M, Vianelli N, Bonifacio M (2018). Efficacy and safety of ruxolitinib in intermediate-1 IPSS risk myelofibrosis patients: results from an independent study. Hematol Oncol.

[15] Polverelli Nicola, Palumbo Giuseppe A., Binotto Gianni, Abruzzese Elisabetta, Benevolo Giulia, Bergamaschi Micaela, Tieghi Alessia, Bonifacio Massimiliano, Breccia Massimo, Catani Lucia, Tiribelli Mario, D'Adda Mariella, Sgherza Nicola, Isidori Alessandro, Cavazzini Francesco, Martino Bruno, Latagliata Roberto, Crugnola Monica, Heidel Florian, Bosi Costanza, Ibatici Adalberto, Soci Francesco, Penna Domenico, Scaffidi Luigi, Aversa Franco, Lemoli Roberto M., Vitolo Umberto, Cuneo Antonio, Russo Domenico, Cavo Michele, Vianelli Nicola, Palandri Francesca (2018). Epidemiology, outcome, and risk factors for infectious complications in myelofibrosis patients receiving ruxolitinib: A multicenter study on 446 patients. Hematological Oncology.

[16] Sant'Antonio E, Bonifacio M, Breccia M, Rumi E (2019). A journey through infectious risk associated with ruxolitinib. Br J Haematol.

[17] Al-Ali HK, Griesshammer M, le Coutre P, Waller CF, Liberati AM, Schafhausen P, Tavares R, Giraldo P, Foltz L, Raanani P, Gupta V, Tannir B, Ronco JP, Ghosh J, Martino B, Vannucchi AM (2016). Safety and efficacy of ruxolitinib in an open-label, multicenter, single-arm phase 3b expanded-access study in patients with myelofibrosis: a snapshot of 1144 patients in the JUMP trial. Haematologica.

[18] Vannucchi AM, Kiladjian JJ, Griesshammer M, Masszi T, Durrant S, Passamonti F, Harrison CN, Pane F, Zachee P, Mesa R, He S, Jones MM, Garrett W, Li J, Pirron U, Habr D, Verstovsek S (2015). Ruxolitinib versus standard therapy for the treatment of polycythemia vera. N Engl J Med.

[19] Lussana F, Cattaneo M, Rambaldi A, Squizzato A (2018). Ruxolitinib-associated infections: a systematic review and meta-analysis. Am J Hematol.

[20] Heine A, Brossart P, Wolf D (2013). Ruxolitinib is a potent immunosuppressive compound: is it time for anti-infective prophylaxis?. Blood.

[21] Porpaczy E, Tripolt S, Hoelbl-Kovacic A, Gisslinger B, Bago-Horvath Z, Casanova-Hevia E, Clappier E, Decker T, Fajmann S, Fux DA, Greiner G, Gueltekin S, Heller G, Herkner H, Hoermann G, Kiladjian JJ, Kolbe T, Kornauth C, Krauth MT, Kralovics R, Muellauer L, Mueller M, Prchal-Murphy M, Putz EM, Raffoux E, Schiefer AI, Schmetterer K, Schneckenleithner C, Simonitsch-Klupp I, Skrabs C, Sperr WR, Staber PB, Strobl B, Valent P, Jaeger U, Gisslinger H, Sexl V (2018). Aggressive B-cell lymphomas in patients with myelofibrosis receiving JAK1/2 inhibitor therapy. Blood.

[22] Barbui T, Ghirardi A, Masciulli A, Carobbio A, Palandri F, Vianelli N, De Stefano V, Betti S, Di Veroli A, Iurlo A, Cattaneo D, Delaini F, Bonifacio M, Scaffidi L, Patriarca A, Rumi E, Casetti IC, Stephenson C, Guglielmelli P, Elli EM, Palova M, Bertolotti L, Erez D, Gomez M, Wille K, Perez-Encinas M, Lunghi F, Angona A, Fox ML, Beggiato E, Benevolo G, Carli G, Cacciola R, McMullin MF, Tieghi A, Recasens V, Marchetti M, Griesshammer M, Alvarez-Larran A, Vannucchi AM, Finazzi G (2019). Second cancer in Philadelphia negative myeloproliferative neoplasms (MPN-K). A nested case-control study. Leukemia.

[23] Polverelli N, Elli E, Abruzzese E, Palumbo GA, Benevolo G, Breccia M, Tiribelli M, Bonifacio M, Tieghi A, Martino B, Sgherza N, D'Adda M, Bergamaschi M, Crugnola M, Cavazzina F, Bosi C, Binotto G, Isidori A, Bartoletti D, Auteri G, Latagliata R, Gandolfi L, Scaffidi L, Cattaneo D, Codeluppi K, Trawinska M, Griguolo D, Cuneo A, Krampera M, Semenzato G, di Raimondo F, Lemoli RM, Cavo M, Vianelli N, Russo D, Iurlo A, Palandri F (2019). B005 Second primary malignancy in myelofibrosis patients treated with ruxolitinib [Abstract]. Haematologica.

[24] Passamonti F, Giorgino T, Mora B, Guglielmelli P, Rumi E, Maffioli M, Rambaldi A, Caramella M, Komrokji R, Gotlib J, Kiladjian JJ, Cervantes F, Devos T, Palandri F, De Stefano V, Ruggeri M, Silver RT, Benevolo G, Albano F, Caramazza D, Merli M, Pietra D, Casalone R, Rotunno G, Barbui T, Cazzola M, Vannucchi AM (2017). A clinical-molecular prognostic model to predict survival in patients with post polycythemia vera and post essential thrombocythemia myelofibrosis. Leukemia.

[25] Tefferi A, Thiele J, Vardiman JW (2009). The 2008 World Health Organization classification system for myeloproliferative neoplasms: order out of chaos. Cancer.

[26] Emanuel RM, Dueck AC, Geyer HL, Kiladjian JJ, Slot S, Zweegman S, te Boekhorst PA, Commandeur S, Schouten HC, Sackmann F, Kerguelen Fuentes A, Hernandez-Maraver D, Pahl HL, Griesshammer M, Stegelmann F, Doehner K, Lehmann T, Bonatz K, Reiter A, Boyer F, Etienne G, Ianotto JC, Ranta D, Roy L, Cahn JY, Harrison CN, Radia D, Muxi P, Maldonado N, Besses C, Cervantes F, Johansson PL, Barbui T, Barosi G, Vannucchi AM, Passamonti F, Andreasson B, Ferrari ML, Rambaldi A, Samuelsson J, Birgegard G, Tefferi A, Mesa RA (2012). Myeloproliferative neoplasm (MPN) symptom assessment form total symptom score: prospective international assessment of an abbreviated symptom burden scoring system among patients with MPNs. J Clin Oncol.

[27] Tefferi A, Cervantes F, Mesa R, Passamonti F, Verstovsek S, Vannucchi AM, Gotlib J, Dupriez B, Pardanani A, Harrison C, Hoffman R, Gisslinger H, Kroger N, Thiele J, Barbui T, Barosi G (2013). Revised response criteria for myelofibrosis: International Working Group-Myeloproliferative Neoplasms Research and Treatment (IWG-MRT) and European LeukemiaNet (ELN) consensus report. Blood.

[28] Tefferi A (2018). Primary myelofibrosis: 2019 update on diagnosis, risk-stratification and management. Am J Hematol.

[29] Kroger NM, Deeg JH, Olavarria E, Niederwieser D, Bacigalupo A, Barbui T, Rambaldi A, Mesa R, Tefferi A, Griesshammer M, Gupta V, Harrison C, Alchalby H, Vannucchi AM, Cervantes F, Robin M, Ditschkowski M, Fauble V, McLornan D, Ballen K, Popat UR, Passamonti F, Rondelli D, Barosi G (2015). Indication and management of allogeneic stem cell transplantation in primary myelofibrosis: a consensus process by an EBMT/ELN international working group. Leukemia.

[30] Marchetti M, Barosi G, Cervantes F, Birgegard G, Griesshammer M, Harrison C, Hehlmann R, Kiladjian JJ, Kroger N, McMullin MF, Passamonti F, Vannucchi A, Barbui T (2017). Which patients with myelofibrosis should receive ruxolitinib therapy? ELN-SIE evidence-based recommendations. Leukemia.

[31] Maschmeyer G, De Greef J, Mellinghoff SC, Nosari A, Thiebaut-Bertrand A, Bergeron A, Franquet T, Blijlevens NMA, Maertens JA, European Conference on Infections in Leukemia (2019). Infections associated with immunotherapeutic and molecular targeted agents in hematology and oncology. A position paper by the European Conference on Infections in Leukemia (ECIL). Leukemia.

[32] Kuykendall AT, Shah S, Talati C, Al Ali N, Sweet K, Padron E, Sallman DA, Lancet JE, List AF, Zuckerman KS, Komrokji RS (2018). Between a rux and a hard place: evaluating salvage treatment and outcomes in myelofibrosis after ruxolitinib discontinuation. Ann Hematol.

[33] Newberry KJ, Patel K, Masarova L, Luthra R, Manshouri T, Jabbour E, Bose P, Daver N, Cortes J, Kantarjian H, Verstovsek S (2017). Clonal evolution and outcomes in myelofibrosis after ruxolitinib discontinuation. Blood.

[34] Pacilli A, Rotunno G, Mannarelli C, Fanelli T, Pancrazzi A, Contini E, Mannelli F, Gesullo F, Bartalucci N, Fattori GC, Paoli C, Vannucchi AM, Guglielmelli P (2018). Mutation landscape in patients with myelofibrosis receiving ruxolitinib or hydroxyurea. Blood Cancer J.

[35] Palandri F, Breccia M, Bonifacio M, Polverelli N, Elli EM, Benevolo G, Tiribelli M, Abruzzese E, Iurlo A, Heidel F, Bergamaschi M, Tieghi A, Crugnola M, Cavazzini F, Binotto G, Isidori A, Sgherza N, Bosi C, Martino B, Latagliata R, Auteri G, Scaffidi L, Griguolo D, Trawinska M, Cattaneo D, Catani L, Krampera M, Vitolo U, Lemoli RM, Cuneo A, Semenzato G, Foà R, Raimondo FD, Bartoletti D, Cavo M, Palumbo GA, Vianelli N (2019). Outcome of patients with myelofibrosis after ruxolitinib discontinuation: role of disease status and treatment strategies in 218 patients [Poster]. Hemasphere.

[36] Masarova L, Bose P, Pemmaraju N, Daver N, Cortes JE, Estrov Z, Kantarjian HM, Verstovsek S (2017) Characteristics and survival of patients with chronic phase myelofibrosis and elevated blasts (5-9%), and the effect of therapy with JAK2 inhibitor ruxolitinib. Am Soc Hematol

[37] Loscocco GG, Mannelli F, Guglielmelli P, Paoli C, Marone I, Cucci R, Coltro G, Sordi B, Albano F, Breccia M, De Stefano V, Finazzi G, Iurlo A, Martino B, Palandri F, Passamonti F, Siragusa S, Mannelli L, Fantoni D, Fazi P, Amadori S, Vignetti M, Barbui T, Vannucchi AM (2019) Italian survey on clinical practice in myeloproliferative neoplasms. A GIMEMA Myeloproliferative Neoplasms Working Party initiative. Am J Hematol. 10.1002/ajh.25555 10.1002/ajh.25555 31179561

