# Supplementary material for: Tracing the decision-making process for myelofibrosis: diagnosis, stratification, and management of ruxolitinib therapy in real-word practice
Source: Ann Hematol. 2019 Dec 12;99(1):65–72. doi: 10.1007/s00277-019-03847-z (PMC6944647; doi:10.1007/s00277-019-03847-z)
Supplement: Supplementary file 1 — Supplementary Table 1. Questionnaire and related answers. (PDF 174 kb) [file 277_2019_3847_MOESM1_ESM.pdf]

ELECTRONIC SUPPLEMENTARY MATERIAL

Annals of Hematology

Tracing the decision-making process for myelofibrosis: Diagnosis, stratification and management of ruxolitinib therapy in real-word practice

Massimo Breccia, Claudia Baratè, Giulia Benevolo, Massimiliano Bonifacio, Elena Maria Elli, Paola Guglielmelli, Margherita Maffioli, Alessandra Malato, Francesco Mendicino, Giuseppe Alberto Palumbo, Novella Pugliese, Elena Rossi, Elisa Rumi, Emanuela Sant'Antonio, Alessandra Ricco, Mario Tiribelli, Francesca Palandri

Dr. Massimo Breccia

Hematology, Department of Precision and Translational Medicine, Policlinico Umberto 1, Sapienza University, Rome, Italy

breccia@bce.uniroma1.it

Supplementary Table 1. Questionnaire and related answers

Questions	Possible answers	Percentages
1. Where do you practice your clinical activity?	a. University	43.75%
	b. Public hospital	56.25%
	c. Private practice	0%
2. How many years of experience do you have in the treatment of chronic myeloproliferative syndromes?	a. <5	12.5%
	b. 5-10	31.25%
	c. >10	56.25%
3. How many newly diagnosed myelofibrosis (MF) patients do you see per year?	a. <5	0%
	b. 5-10	43.75%
	c. >10	56.25%
4. You have experience in treating patients with:	a. Only MF	0%
	b. MF and PV	0%
	c. MF and ET	0%
	d. MF, PV, ET	100%
5. Do you use the IPSS score at baseline?	a. Yes	100%
	b. No	0%
6. Do you use the MIPSS or MIPSS-70 score at baseline?	a. Yes	25%
	b. No	75%
7. Do you use the DIPSS score during treatment?	a. Yes	93.75%
	b. No	6.25%
8. Do you use the MYSEC-PM score for secondary MF?	a. Yes	81.25%
	b. No	18.75%
9. When do you decide to re-evaluate with bone marrow biopsy a patient with PV in case of MF suspected evolution?	a. In the case of development/progression of splenomegaly	25%
	b. In the case of systemic symptoms not present before	0%
	c. If phlebotomies are no longer needed	6.25%
	d. When at least two of the previous criteria are met	68.75%
10. When do you decide to re-evaluate with bone marrow biopsy a patient with ET in case of MF suspected evolution?	a. In the case of development of splenomegaly	18.75%
	b. In the case of systemic symptoms not present before	0%
	c. If cytoreduction is no longer needed	12.5%
	d. When at least two of the previous criteria are met	68.75%
11. How often do you regularly visit a patient with IPSS low / intermediate-1 risk?	a. Once a month	0%
	b. Once every 3 months	62.5%
	c. Once every 6 months	37.5%

	d. Once a year	0%
12. How often do you regularly visit a patient with IPSS intermediate-2/high risk? (excluding transfusion-only access)	a. Once a month<br>b. Once every 3 months<br>c. Once every 6 months<br>d. Once a year	50%<br>43.75%<br>6.25%<br>0%
13. When do you treat a patient with low IPSS risk?	a. Only in case of IPSS int-2 progression<br>b. If splenomegaly is > 5 cm<br>c. If symptoms are present<br>d. I do not treat patients with IPPS low risk but I only observe them	6.25%<br>31.25%<br>37.5%<br>25%
14. When do you treat a patient with intermediate-1 IPSS risk?	a. If splenomegaly is > 5 cm<br>b. If symptomatic (TSS > 40 or very high single item score)<br>c. a+b<br>d. I do not treat patients with IPSS int-1 risk	6.25%<br>0%<br>93.75%<br>0%
15. Do you have the opportunity to test for non-driver mutation in your center?	a. Yes<br>b. No	37.5%<br>62.5%
16. Do you test non-driver mutation (i.e. ASXL1, etc) in your patients?	a. No<br>b. Yes, always also for patients positive for driver mutations<br>c. Yes, only for young patients (< 50 years)<br>d. Yes, but only for intermediate-1 risk patients to decide the best therapeutic transplant strategy	25%<br>12.5%<br>25%<br>37.5%
17. Do you perform cytogenetic analysis in MF patients?	a. Yes, routinely for all<br>b. Never<br>c. Only for patients <50 years	81.25%<br>0%<br>18.75%
18. How do you routinely evaluate splenomegaly?	a. Manually<br>b. With ultrasound<br>c. With abdominal CT/NMR	56.25%<br>43.75%<br>0%
19. For an int-2/high risk patient, do you use first-line ruxolitinib immediately?	a. No, only after hydroxyurea<br>b. Yes, immediately	12.5%<br>87,5%
20. Before starting ruxolitinib, in view of the possible reactivation of hepatotropic viruses, do you check for hepatitis serology?	a. Yes, but only for HBsAg<br>b. Yes, I check for full panel and also HBV-DNA<br>c. I do not perform for all patients but only in patients with previous positivity	12.5%<br>87.5%<br>0%
21. In case of seropositive patient for hepatitis B, do you perform a prophylaxis?	a. Yes<br>b. No	87.5%<br>12.5%
22. Do you screen for previous TBC infection before to start with ruxolitinib?	a. Yes, always<br>b. No, never<br>c. Yes, only in case of a past medical history	93.75%<br>0%<br>6.25%
23. For TBC test, what do you mostly use?	a. QuantiFERON-TB Gold<br>b. Mantoux intradermal test<br>c. CT scan	81.25%<br>12.5%<br>6.25%
24. Before starting treatment with ruxolitinib, do you perform viral serology for herpetic viruses (CMV, HSV1-2, EBV, VZV)?	a. Never<br>b. Yes, always<br>c. Only for some frequent viruses	50%<br>37.5%<br>12.5%
25. Do you perform antiviral prophylaxis during ruxolitinib treatment?	a. No, never<br>b. Yes, always<br>c. Yes, but only in patients with a history of previous infections<br>d. Yes, but only in secondary prophylaxis after one or more episodes of herpes zoster reactivation during treatment	6,25%<br>0%<br>18.75%<br>75%
26. How do you behave in case of infection during ruxolitinib?	a. Reduce temporarily the dose<br>b. Discontinue the drug and then resume at the same dose after the resolution of the event<br>c. Definitely discontinue the drug<br>d. Discontinue the drug and the resume	62.5%<br>37.5%<br>0%<br>0%
27. How do you schedule the monitoring visits starting with ruxolitinib?	a. Once a week for the first 2-3 months<br>b. Every 15 days<br>c. Once a month<br>d. Initially weekly and then depends on hematologic toxicity	12.5%<br>18.75%<br>25%<br>43.75%
28. In case of hematologic toxicity (grade 3 anemia):	a. Discontinue the treatment<br>b. Reduce the dosage	0%<br>18.75%

	c. Transfuse the patient (even naïve) and continue with the same dosage	81.25%
29. Do you use erythropoietin during treatment with ruxolitinib in case of anemia?	a. Never<br>b. Yes, always<br>c. Yes, but only in patients with serum EPO dosage lower than then normal	12.5%<br>25%<br>62.5%
30. In case of hematological toxicity (grade 3 thrombocytopenia):	a. Discontinue the treatment<br>b. Reduce the dosage<br>c. Transfuse the patient (even naïve) and continue with the same dosage	18.75%<br>81.25%<br>0%
31. In case of toxicity and need for interruption, how do you carry out the suspension?	a. Gradually reduce and then discontinue<br>b. Discontinue the drug abruptly	87.5%<br>12.5%
32. After how long on average patients report a resolution / improvement in their symptoms?	a. 1-2 weeks from the start of treatment<br>b. 3-4 weeks from the start of treatment	62.5%<br>37.5%
33. Do you use the MPN10 instrument to quantify symptoms?	a. Yes<br>b. No	75%<br>25%
34. Based on your experience, how many patients with low / intermediate-1 risk are symptomatic?	a. 30-40%<br>b. Less than 30%<br>c. More than 50%	25%<br>62.5%<br>12.5%
35. How do you rate a splenic response to ruxolitinib?	a. Consider the COMFORT studies criteria (> 35% reduction in basal volume)<br>b. Consider a reduction > 50% of the spleen length<br>c. According to 2013 IWG criteria	25%<br>31.25%<br>43.75%
36. Do you consider a dose reduction in the long-term responder?	a. Never<br>b. Yes, always<br>c. Yes, but only if the patient has concomitant toxicity	25%<br>31.25%<br>43.75%
37. When do you consider a patient in therapeutic failure after treatment with ruxolitinib?	a. In case of absolute lack of splenic response and symptoms<br>b. Worsening of general conditions (increase in symptoms and splenomegaly) during treatment<br>c. There are no failure criteria and therefore I continue the treatment even if the patient has responded only from a symptomatic point of view	18.75%<br>43.75%<br>37.5%
38. Do you consider re-evaluating bone biopsy during treatment?	a. Yes, in patients with complete response<br>b. No<br>c. Yes, after at least one year of treatment	50%<br>18.75%<br>31.25%
39. Do you consider possible pharmacological associations in patients with non-optimal response?	a. No<br>b. Yes	81.25%<br>18.75%
40. What is the drug you most frequently associate with ruxolitinib?	a. Erythropoietin<br>b. Hydroxyurea<br>c. Anabolic<br>d. Vitamins	25%<br>43.75%<br>6.25%<br>25%
41. Do you perform a transplant assessment in patients under the age of 70 (more than one answer is possible)?	a. At baseline, if IPSS Intermediate-2 /High<br>b. At progression, if DIPSS Intermediate-2 / High<br>c. If IPSS / DIPSS Intermediate-1 in the presence of factors that negatively impact the prognosis<br>d. Only if the patient does not reach or lose a response to ongoing treatments and in the absence of therapeutic alternatives	93.75%<br>56.25%<br>81.25%<br>25%
